# Recovery of Cognitive Functioning in Alcoholics: The Relationship to Treatment

**Published:** 1995

**Authors:** Mark S. Goldman

**Affiliations:** Mark S. Goldman, Ph.D., is Distinguished Research Professor, director of the Alcohol and Substance Use Research Institute, and director of training in clinical psychology, Department of Psychology, University of South Florida, Tampa, Florida

**Keywords:** AOD impairment, cognitive process, treatment program, treatment method, treatment outcome, AODD (alcohol and other drug use disorders) recovery

## Abstract

Alcoholics’ successful recovery depends on their regaining cognitive functioning. Although their cognitive deficits often are subtle and improve with a period of abstinence from alcohol, they can hamper the effectiveness of treatment programs. If patients cannot comprehend the information imparted during therapy, they may not be able to use treatment strategies successfully in “real world” challenges. Cognitive recovery can be enhanced using strategies such as repeated mental exercises. Adding such practice to treatment regimens could improve some alcoholics’ chances of recovering successfully.

A quick review of this issue of *Alcohol Health & Research World* reveals the impact of chronic excessive alcohol use on cognitive functioning. The most severe impairments are the profound memory dysfunction caused by Wernicke-Korsakoff syndrome (for a definition of this syndrome, see the glossary, pp. 136–137) or the more global intellectual deterioration (including memory impairment) of alcoholic dementia (i.e., general loss of memory functioning, judgment, and abstract thinking). Even among people admitted to alcoholism treatment facilities^1^ who show no dramatic cognitive impairment, between 75 and 100 percent of the cases (depending on the samples and measures used) perform below normal for their age group on sensitive tests of cognitive functioning (McCrady and Smith 1986).

Until recently, researchers often have attributed the many levels and types of cognitive deficits seen among alcoholics to differing forms of alcohol-related damage to the drinkers’ neuroanatomy (i.e., the brain’s various parts) and neurophysiology (i.e., the functions of these parts). Researchers considered such differences in impairment to be responsible for any performance discrepancies observed among alcoholics. In addition, researchers attributed Wernicke-Korsakoff amnesia to thiamine deficiency and to the lesions accompanying this syndrome in a variety of brain structures, such as the diencephalon, mammillary bodies, and basal fore-brain, which are associated with memory functions (for definitions and further descriptions, see the diagram of the brain on p. 137). Deficits in problem-solving, abstracting (i.e., ascertaining the principles or rules that govern a particular task), and shifting of sets (i.e., recognizing the need for using new rules when the previous rules no longer apply) were associated with frontal lobe damage caused by alcohol consumption or related factors. Some visuospatial impairments were connected with a wasting away (i.e., atrophy) of the frontal and/or right hemisphere. In recent years, however, investigators have used more sophisticated brain imaging and cerebral blood flow techniques and found similar neuroanatomical damage in all alcoholics, including those whose deficits are detectable only on sensitive behavioral tests. Thus, differences in cognitive capacity among alcoholics cannot be attributed exclusively to differences in their neurophysiology.

How similar forms of damage to the nervous system can result in differing behavioral consequences, including cognitive deficits, in different alcoholics remains unclear. The behavioral changes that researchers observe may result from the general effects of alcohol-related toxicity and other factors (e.g., head injury, liver damage, psychiatric factors, or neurochemical abnormalities) combined with individual differences, such as age and drinking history (Bowden 1990; National Institute on Alcohol Abuse and Alcoholism 1993). Some researchers also have suggested that a portion of the impairments are present in people with a family history of alcoholism even before they begin consuming alcohol (the accumulated evidence for preexisting dysfunction has been mixed, however; see Drake et al. 1995).

Alcohol researchers do not know whether cognitive impairments impede alcoholism treatment. Can a cognitively impaired alcoholic readily absorb all the information that is usually imparted during treatment? Do these deficits make the necessary adjustments and adaptations to a “dry” (i.e., nondrinking) way of life more difficult? If so, can anything be done to help the alcoholic recover from these deficits? This article discusses the partial answers to these questions, first reviewing the course of recovery from alcohol-related deficits, then considering how these deficits may affect treatment outcomes. It concludes by reviewing research on ways to improve treatment outcome by facilitating cognitive recovery.

## The Nature of the Deficits and How They Are Determined

Test findings from a wide group of studies show that alcoholics are remarkably free of impairment of general intelligence. Their cognitive deficits are more consistently revealed using specific tests of abstract reasoning and visual perception. In addition, alcoholics have not consistently shown learning and memory deficits despite the fact that more severe versions of these impairments are symptoms of Wernicke-Korsakoff syndrome (see Parsons et al. 1987).

These descriptions of alcohol-induced deficits derive primarily from the researchers’ intuitive analyses of what the tests seem to measure, such as abstracting ability or memory. Some investigators use more sophisticated strategies based on cognitive psychology to better understand the nature of the cognitive dysfunctions. For example, Parsons (1987) and coworkers noticed that alcoholics appear to change a strategy (that may be correct) before it has been sufficiently tested or to continue using ineffective approaches even after it is obvious that they are inadequate. On difficult verbal learning tasks, Butters and Granholm (1987) have suggested that cognitive deficits stem from the inadequate encoding strategies alcoholics use when storing information rather than from a specific inability to learn or remember. In other words, correct information may be placed in a file drawer, but an inadequate label on the file might make retrieval of this information difficult.

Cognitive psychology’s techniques thus attempt to uncover impairments in general cognitive processes that may affect many other brain functions. Statistical research supports the possibility that the more specific deficits in abstracting ability, visuoperception, learning, and memory may be caused by more general and pervasive information-processing deficits. Studies indicate that a single underlying process may be associated with most, if not all, the observed deficits on specific tests.

Goldman (1987, 1990) has suggested that the tests most sensitive to alcohol-related cognitive dysfunction have several characteristics in common. They all present stimulus material that the patient has not previously experienced. They require that multiple kinds of information be integrated (e.g., learning to connect a name with a face). And, in most cases, they require that the information be processed rapidly. These challenges require a process traditionally called “attention”; the more recently used term is “controlled.” (In fact, a distinguishing characteristic of Wernicke-Korsakoff syndrome is impaired controlled memory processes, whereas implicit memory—using remembered information or a newly learned skill without being aware of when or how it was learned—remains relatively intact. For further discussion, see the article by Ingle and Weingartner, pp. 155–158.)

The capacity to deal with new situations that demand the processing of multiple sources of information underlies humans’ ability to adapt to changing circumstances. Recovering alcoholics require such adaptability to change from a lifestyle that includes continual drinking to one that involves no drinking. Hence, alcoholics may be deficient in exactly those cognitive capabilities they need the most to recover successfully from alcoholism.

## Time-Dependent Recovery

When alcoholics cease continual heavy drinking (e.g., as a result of admission to detoxification programs), they typically experience a period of acute withdrawal that may last a few days. During this time, they feel ill and frequently show poor performance on most cognitive tests, probably as much a result of a general sense of malaise as any other factor. It is not surprising, therefore, that they improve on these tests after the acute withdrawal phase. Beyond this relatively brief period, however, real improvement may be observed as time passes. The rate of improvement and the ultimate level of functioning the alcoholic reaches vary with the type of cognitive processing involved in completing a task and with the age of the alcoholic. Sometimes complete recovery of cognitive functioning can take weeks, or even months or years. In some instances, the alcoholic never completely recovers.

### Determining Recovery Patterns

To see how alcoholics’ performances change over time after they cease drinking, it is necessary to measure their performance on at least two occasions over a specific length of time. Recovery may not progress at a steady pace, so it is best to measure performance on more than two occasions. However, people improve their performance of most behaviors after they practice those behaviors.^2^ Therefore, if an alcoholic improves after repeatedly performing a particular task, the improvement may be the result of either true (generalized) cognitive recovery over time or only increased familiarity and practice with the specific instrument used to measure the targeted behavior.

### Controlling for the Effects of Practice

Two general approaches have been used to separate recovery from the effects of practice. In one case, each matched group of alcoholics is tested for the first time at different time lags after stopping drinking, followed by repeat testings also at different times. For example, group one may be tested at weeks 1, 2, and 3 after drinking has stopped, whereas group two may be tested at weeks 2, 3, and 4. This way, the effects of practice on the tests can be separated from recovery that occurs over time. If at the first test, group two performs better than group one, then time-dependent recovery is evident. Repeat testings are necessary to ensure that differences between the supposedly matched groups are not the result of unintended discrepancies between the groups (e.g., differences in premorbid intelligence). In the other approach, non-alcoholics (usually matched with the alcoholics in education and sociodemographic status) are given the same series of tests as the alcoholics to determine what improvement on the tests would be if only practice (and no time-dependent recovery) was occurring. The difference in the rate of improvement between the alcoholics and the nonalcoholics is then an indication of the “true” recovery of cognitive functioning.

### Patterns of Time-Dependent Recovery

When these methodological issues are taken into account and the recovery literature is considered, the following patterns of time-dependent cognitive recovery emerge (see Goldman 1987, 1990). First, some cognitive capacities seem relatively unimpaired, even early in detoxification, as long as the general malaise of the first few days of abstinence is past. Gross IQ, as measured primarily by verbal tests that draw upon prior knowledge, falls into this category. This means, for example, that the vocabulary levels of very recently detoxified alcoholics are about the same as they were prior to and after recovery from the acute alcoholic episode that brought them into detoxification. In contrast, any task that requires processing new information, abstracting, or problem-solving, whether verbal or visuoperceptual, still is impaired during the first week or two after drinking ceases. Some sensorimotor functions (e.g., sensitivity to touch) also may be deficient during this period. Other factors, such as age and drinking history, also affect time-dependent recovery.

#### Age

Two to 3 weeks after alcoholics stop drinking, they show considerable recovery in most verbal processing cognitive functions; these areas may even return to normal functioning levels. At this point, however, the recovery paths of alcoholic subgroups diverge, based primarily on their age. Younger alcoholics (those under age 40) show substantial recovery of all cognitive functions; only the most demanding tests detect residual deficits. For older alcoholics, the picture differs. Although their performance on cognitive tests may continue to improve, deficits can be observed on visuoperceptual and problem-solving tasks for much longer periods of time, even as long as many months or years. In certain studies examining deficits in short-term memory, visuospatial functioning, and attention among older alcoholics, problems have been identified even after 5 years (Brandt et al. 1983).

#### Drinking History

Most studies have not found that an alcoholic’s drinking history relates significantly to the speed or extent of recovery. Alcoholics with more years of heavy or problem drinking are not more likely to have more lasting impairment than are those with fewer years. This finding is counterintuitive, and the reasons for it are not entirely clear. The brains of people with shorter drinking histories may be more resilient physically or may better carry out neurophysiological adjustments. Or, up to a certain number of drinking years, alcoholics may be able to learn to compensate for underlying neurological damage to produce unimpaired behavior (e.g., by performing a task a different way). Perhaps a dysfunctional performance only appears after excessive drinking has gone on for a certain length of time, producing a threshold above which cognitive impairments become observable. Or it may be that some as-yet-undetermined process is at work.

It is clear, however, that a return to alcohol use, even at reduced levels, after some period of sobriety sets back the recovery process regardless of drinking history. Very recent findings further indicate that the effect of drinking resumption may be more debilitating for alcoholics who also have family histories of alcoholism, although such alcoholics apparently recover just as well as alcoholics without such histories if they maintain abstinence (Drake et al. 1995).

## Experience-Dependent Recovery

In a series of studies performed over the last 20 years, Goldman (1990) found that cognitive recovery does not result only from some intrinsic neurophysiological healing process but can be influenced by environmental factors as well. These environmental factors may be likened to physical exercise, but in this case, the “exercise” involves cognitive stimulation. Recovery seems to be accelerated if newly abstinent subjects are asked to “use their heads” at a level that is equal to, or slightly beyond, their current level of functioning. This experience-dependent recovery may happen spontaneously because of naturally occurring events, as when a job requires that a task be performed repeatedly. Alternatively, recovery can be facilitated by planned cognitive activities, such as repeating mental exercises, similar to the use of physical therapy to recover after a sports injury. In a loose sense, the cognitive “switchboard” of the alcoholic appears impaired but apparently can be stimulated to more efficient activity by the repetition of appropriate cognitive demands.

Experience-dependent recovery is by no means unique to alcoholism research. Inducing recovery from brain damage by manipulating environmental variables has been seen before in both animal and human research (see Rose and Johnson 1992). Evidence exists now that such recovery is not only a consequence of the subject’s adjusting behavior to learn a new method of performing a task. Indeed, studies using a variety of designs to examine the effects of environmental changes on neurological functioning have found performance enhancement coupled with actual changes in the nervous system (for further information, see Rose and Johnson 1992).

### Experience-Dependent Techniques for Inducing Cognitive Recovery

#### Practice

The basic strategy for influencing an alcoholic’s cognitive recovery has been to repeatedly administer tests that demonstrate the subject’s impairment. This procedure is nothing more than practice, discussed earlier as a possible experimental confound in time-dependent recovery studies. Reexamined in this new context, however, practice does more than facilitate trivial performance improvement on a specific test. If a particular cognitive test is uniquely sensitive to some underlying neurological damage, the improvement caused by repeated performance of that test is not trivial. No one would consider insignificant an increase in the strength of an atrophied muscle as a result of an exercise regimen; this process would be called rehabilitation. Similarly, the improvement in performance resulting from practice on one cognitive test uniquely sensitive to some underlying neurological damage should transfer to improved performance on other tests that seem based on a similar cognitive function.

In the early studies of experience-dependent recovery (Forsberg and Goldman 1985), subjects practiced one version of a particular test and then were tested on another version of the same test to demonstrate the transferability of their performance improvement. In more recent studies (Forsberg and Goldman 1987), practice on demanding visuospatial learning tests has resulted in performance improvements on a wide variety of other cognitive tests, but only if the tests were presented within the same sensory modality. For example, practice on some visually presented tests resulted in improved performance on other visually presented tests but did not seem to improve performance on tests that depended primarily on touch. Nevertheless, the broad transfer of performance improvement suggests that providing practice for controlled, attention-demanding cognitive tasks could enhance the impaired subjects’ cognitive capabilities in other areas. In younger people, whose improvement could occur spontaneously over time, cognitive improvement seemed to be accelerated by practicing. In older alcoholic subjects, practicing helped increase their cognitive functioning, even on tests that would have revealed impairment for a much longer time if they had not practiced. Cognitive performance did not always improve to normal levels as a result of practicing, but it did improve significantly (Goldman 1987).

#### Other Strategies

Goldman and colleagues (1987; 1990) investigated whether other experience-dependent strategies to induce recovery might be superior to simple repetitive practice. To this end, they broke a complex task into its component parts and trained subjects to perform these components so that the retraining process was easier and more accessible to people who might be frustrated by their cognitive dysfunction. Although subjects recovered after this strategy beyond what they would have with no training, the strategy was no better than simple practice. Apparently, alcoholics generally were not impaired to the extent that they required a more elemental strategy than that of practice (as severely brain-damaged subjects might).

A second strategy depended on practicing a task that was specifically designed to require attention and effortful cognitive functioning. As seen in the first strategy, recovery using these techniques was approximately the same as recovery with simple practice on more traditional cognitive (neuropsychological) tests. This finding was consistent with the theory that a basic cognitive deficit in alcoholics is in the brain system(s) that control(s) effortful processing and integration of multiple sources of information.

### Implications of Cognitive Recovery

The general improvement seen in alcoholics’ cognitive functioning after experience-dependent recovery raises two fundamental questions with implications for successful treatment. First, does the cognitive improvement extend to behaviors that are directly associated with treatment (e.g., communication skills)? Second, can cognitive rehabilitation strategies be used deliberately with alcoholics to improve their treatment outcome? The following section reviews the findings relating cognitive functioning to treatment outcome in general. Unless impaired cognitive functioning prevents or retards effective treatment outcome, improved cognitive functioning would not affect how an alcoholic responds to treatment.

## Does Cognitive Status Affect Treatment Outcome?

As noted earlier, alcoholics’ cognitive deficits most often are subtle. Whether deficits of this type have any relationship with treatment outcome is a question that must be answered with empirical research. To date, some research does indicate that cognitive functioning (or dysfunctioning) relates to various aspects of treatment, including treatment outcome. For example, different studies have shown that less cognitively impaired alcoholics are more likely to attend outpatient treatment, to complete a treatment program, to be rated by treatment personnel as having a better prognosis, and actually to have a better outcome. Other studies have found that cognitive measures predict how long after treatment a patient will resume drinking and the chances of a patient remaining abstinent for more than 6 months following treatment discharge. Alcoholics with better cognitive functioning are more likely to have full-time employment and a higher monthly income at followup than are more cognitively impaired alcoholics (see Goldman 1990 for a review of specific studies).

On the other hand, some researchers have reported the relationship between cognitive deficits and treatment success to be modest at best or even inverse. They note that adding indicators of patients’ cognitive status to statistical analyses does not increase the accuracy of the treatment outcome predictions that result from using only basic sociodemographic variables. Other researchers have urged caution before any adjustments are made to existing treatment programs that are based on what they consider to be an uncertain relationship (see Donovan et al. 1987, Goldman 1990, and Goldstein 1987 for more extensive discussions of the inconsistencies between these studies).

### The Bases for the Inconsistent Findings

To understand why findings on cognitive impairment have been mixed, it is necessary to appreciate that adequate cognitive functioning does not, by itself, ensure a better treatment outcome. It does provide a foundation on which other treatment-related factors may operate.^3^ The capacity to learn the kinds of skills and information that are taught by most treatment programs may be increased if the patient’s thinking and learning mechanisms are intact. At least four factors may be responsible for the lack of consistent observations on the relationship between cognitive functioning and treatment outcome.

First, the cognitive tests used in the studies described above are not necessarily those best suited (most valid) for detecting the aspects of dysfunction closely related to treatment outcome and general life functioning. These tests were originally selected because they were sensitive to brain damage caused by stroke, tumors, head injuries, neurological diseases, and other physical conditions and not because they could assess optimally the wide range of behaviors needed in day-to-day living. Some neuropsychologists (Heaton and Pendelton 1981) suggest the need for tests that are similar to daily activities. For example, when a test based on knowledge of familiar advertising used in magazines was used to assess cognitive functioning in alcoholics, this test proved more statistically predictive of treatment outcome than did entire batteries of standard cognitive tests (Sussman et al. 1986).

Second, some research suggests that many current treatment modalities only minimally impact the factors influencing an alcoholic to drink. Therefore, it would not matter whether the cognitively impaired alcoholic could or could not learn the behavior taught by the treatment program. If a method for teaching algebra is unclear and ineffective, both highly intelligent and less intelligent children will fail to learn, reducing the observed relationship between intelligence and learning algebra. If alcoholism treatments are ineffective, reduced relationships between cognitive impairment and positive treatment outcome may only reflect the ability of alcoholics to recover on their own without the benefit of treatment-acquired coping strategies. Treatments themselves must be improved, and/or they must be matched to the functional cognitive level of the alcoholic before the true importance of differences in cognitive functioning can be identified and evaluated. For example, two recent reports on a patient-treatment matching study (Cooney et al. 1991; Kadden et al. 1989) provide somewhat unintended evidence for the importance of matching treatment complexity to patients’ cognitive resources. Cognitively impaired patients did better in loosely structured interactional group therapy than in highly structured behavioral coping skills training (the investigators originally had indicated that the structured training should offer an advantage for cognitively impaired alcoholics). Perhaps this result is not so surprising, however, when the large amount of information that must be acquired during coping skills training is compared with the considerably lighter informational demands of interactional therapy.

Third, in the first weeks and months after they stop drinking, alcoholics face a variety of environments, ranging from the very supportive to the very harsh. The more demanding the environment, the greater the recovering alcoholic’s need will be for cognitive resources. The relationship between an alcoholic’s cognitive status and treatment outcome will become clear only when the alcoholic experiences posttreatment events, such as finding and learning a new type of job, that will challenge the alcoholic’s cognitive capacity.

Finally, cognitive functioning is only one among many influences that may affect treatment outcome. Motivation, the availability of social support networks, employment opportunities, comorbid psychiatric disorders, and numerous other factors also may play a role in how the alcoholic responds to treatment.

On the other hand, research reports may occasionally obscure the impact of cognitive deficits because the deficits interact with or overlap other treatment-related factors. For example, measures that predict treatment outcome—such as whether a person is able to perform an intellectually demanding job—contain components of cognitive ability. These predictors could be considered both sociodemographic factors and factors resulting from the extent of a person’s cognitive impairment. Thus, the idea that cognitive impairment may not add to the predictive accuracy of sociodemographic factors on these outcomes does not mean that cognitive deficits have no effect on job performance. These apparently different indices may be measuring the same thing, and the results from one set may mask the value of results from the other set.

## The Impact of Cognitive Deficits on Treatment Outcome

It is possible that even subtle cognitive deficits could affect how alcoholics seek and participate in treatment and resume normal lives in the weeks and months after they stop drinking. Three examples of different types of deficits and their impact on elements of treatment are presented below.

First, treatment professionals understand “classic alcoholic denial” as a kind of psychological avoidance or evasion of unpleasant reality. Part of this denial, however, may result instead from the alcoholic’s limited ability to process the full range of available information about his or her drinking problem and a behavioral inflexibility in making necessary changes in stopping the drinking. If denial is viewed as a part of the temporary brain damage caused by alcohol’s toxic effects rather than as a refusal to accept responsibility, different treatment approaches may be indicated for engaging the alcoholic in treatment other than the currently popular confrontational methods. These new approaches are more consistent with newer recommendations to avoid confrontational strategies and instead use strategies that increase motivation (Miller and Rollnick 1991).

Second, almost all treatment approaches depend, at some fundamental level, on interpersonal communication skills. Cognitive confusion may impede alcoholics’ ability to effectively express their own thoughts and feelings as well as to clearly receive communications from treatment personnel. All aspects of treatment may be affected by this difficulty.

Third, the essence of all treatment is the need for change—change in how one views the world and interacts with other people when not drinking and change in many routine habits. Unfortunately, the most frequent common denominator of cognitive impairment, including that which results from alcoholism, is the lessening of adaptability and flexibility. Even a quick review of Alcoholics Anonymous’ (AA’s) 12-step philosophy reveals how much abstract thinking, concentration, and memory are required to absorb this material cognitively and apply it to maintaining a new lifestyle (e.g., recognizing that one is “powerless over alcohol,” taking a “searching and fearless moral inventory,” and listing “all persons...harmed”; AA World Services 1978). Similar cognitive demands arise in connection with most cognitive behavioral treatments and in treatments that include learning information about how alcohol affects the body and the mind. Not only must the alcoholic make changes as part of treatment, but the new behavioral repertoire learned also must be implemented in constantly varying daily situations. The alcoholic must be able to recognize a potentially problematic situation, resist old maladaptive responses, and implement new behaviors that may be far from thoroughly learned.

## Improving Treatment Outcomes by Facilitating Cognitive Recovery

**Figure 1:**
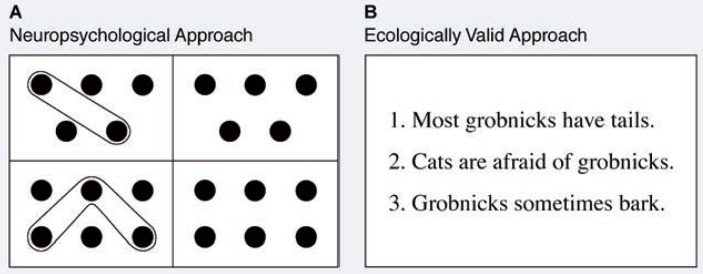
Alcoholics practice cognitive rehearsal tasks to improve their cognitive functioning. Two examples of these tasks (as presented in workbook format in the study by Roehrich and Goldman [1993]) include the neuropsychological approach (A), in which the patient must look at the dot designs in the boxes at the left and then make the same designs in the boxes at the right, and the ecologically valid approach, or word-finding task (B), in which the patient must guess the meaning of a missing word (represented by a nonsense word) that appears in several sentences.

As was demonstrated in the previous section, alcoholics may not benefit from certain aspects of treatment because of their cognitive deficits. As a result, alcoholics with greater initial impairment would have a better chance of recovery from alcoholism if their cognitive improvement could be accelerated and brought to levels approaching normal before they entered treatment. In a recent study, Roehrich and Goldman (1993) found that they could use experience-dependent recovery strategies to help accomplish these ends. The procedure essentially was the same as that used in earlier experience-dependent recovery research, with impaired alcoholics beginning a sequence of repeated rehearsals of cognitive tasks shortly after they completed detoxification (figure 1). One significant change from prior studies was that the researchers gave the tasks to the participants in self-administered workbooks, rather than being administered by assistants in a face-to-face format. If cognitive improvements could be observed in this format, the remediation procedure could be far less labor intensive and costly for actual clinical settings.

An even more critical change was that the alcoholic patients’ ability to learn and implement a treatment component became a criterion for judging whether the alcoholic had successfully benefited from the cognitive rehabilitation program. In their study, Roehrich and Goldman (1993) used relapse prevention training as the treatment component. They implemented this training in the latter phases of the cognitive rehabilitation program. Four remediation strategies were compared, with a different group assigned to each intervention. The strategies included practice on standard cognitive (i.e., neuropsychological) tasks, practice on ecologically relevant tasks (figure 1), practice on placebo tasks (which required only automatic verbal responses), and no practice at all. Results showed that the remediation strategies that involved real tests were equally effective in helping alcoholics learn the relapse prevention material; they also were superior to both the placebo and no treatment groups. Long-term treatment outcome must await future research.

## Conclusions and Final Treatment Recommendations

Although the application of what is known about cognitive recovery to alcoholism treatment is in its early stages, several recommendations can be made that then must be tested with appropriate research designs.

For example, many studies have demonstrated the profound cognitive deficits frequently seen in some alcoholics during withdrawal. Cognitive status, therefore, could be assessed routinely to guide treatment planning. For cognitively impaired alcoholics, the use of treatment components that demand heavy cognitive processing (these would include most current treatment methods) could be delayed until at least 1 to 2 weeks after the patients cease drinking. During this time, the treatment emphasis should be on assisting the recovering alcoholic to avoid alcohol through a brief inpatient stay or by close monitoring on an outpatient basis by family members or friends (this approach is similar to some strategies used by traditional alcoholism treatment programs). After alcoholics have passed through this critical period, treatment components may be introduced in a systematic fashion, beginning with the less cognitively demanding and progressing to the more demanding. Attention to the therapy’s cognitive demands on the patient and to the cognitive needs of each patient (i.e., those required by the patient to cope in his or her environment) probably should continue well beyond traditional treatment periods, into the aftercare phase.

In addition, information presented to patients should be concrete rather than abstract; active strategies that emphasize practice may be used. Also, treatment professionals must not depend on alcoholics being able to demonstrate “quick thinking” in high-risk situations that may trigger drinking. Alcoholics must be able to practice with specific behaviors in treatment that reduce risk until these behaviors are as automatic as possible. These suggestions are in keeping with relapse prevention training.

Finally, facilitating the alcoholics’ cognitive recovery using experience-dependent procedures may help reduce the risk of relapse. These methods have shown promise in preliminary studies and warrant further research.

## Footnotes

1 In general, the patients described in this article as having been admitted to alcoholism treatment facilities meet the criteria for alcohol dependence listed in the American Psychiatric Association’s *Diagnostic and Statistical Manual of Mental Disorders, Fourth Edition* (DSM–IV).

2 A total failure to improve with practice would be a sign of rather severe brain dysfunction. As noted earlier, even the most severely impaired alcoholics typically show some recovery of function when they cease drinking.

3 In statistical terms, adequate cognitive functioning serves as a moderator.
